# Foxp3 Molecular Dynamics in Treg in Juvenile Idiopathic Arthritis

**DOI:** 10.3389/fimmu.2018.02273

**Published:** 2018-10-02

**Authors:** Alastair Copland, David Bending

**Affiliations:** Institute of Immunology and Immunotherapy, College of Medical and Dental Sciences, University of Birmingham, Birmingham, United Kingdom

**Keywords:** Foxp3, juvenile idiopathic arthritis (JIA), transcriptional dynamics, Tocky, Treg

## Abstract

Since the identification of the regulatory T-cell (Treg)-associated transcription factor Foxp3, there have been intensive research efforts to understand its biology and roles in maintaining immune homeostasis. It is well established that thymic selection of a repertoire of self-reactive Foxp3^+^ T-cells provides an essential mechanism to minimize reactions to self-antigens in the periphery, and thus aid in the prevention of autoimmunity. It is clear from both genetic and immunological analyses of juvenile idiopathic arthritis (JIA) patients that T-cells have a strong role to play in both the initiation and propagation of disease. The current paradigm is to view autoimmunity as a consequence of an imbalance between inflammatory and immunoregulatory mechanisms. This view has led to the assigning of cells and inflammatory mediators to different classes based on their assumed pro- or anti-inflammatory roles. This is typically reported as ratios of effector T-cells to Treg cells. Problematically, many analyses are based on static “snapshots-in-time,” even though both mouse models and human patient studies have highlighted the dynamic nature of Foxp3^+^ T-cells *in vivo*, which can exhibit plasticity and time-dependent functional states. In this review, we discuss the role of Foxp3 dynamics in the control of T-cell responses in childhood arthritis, by reviewing evidence in humans and relevant mouse models of inflammatory disease. Whilst the cellular dynamics of Treg have been well evaluated—leading to standard data outputs such as frequency, quantity and quality (often assessed by *in vitro* suppressive capacity)—we discuss how recent insights into the molecular dynamics of *Foxp3* transcription and its post-translational control may open up tantalizing new avenues for immunotherapies to treat autoimmune arthritis.

## Aims and scope

The aim of this article is to provide an overview of the literature reporting Foxp3^+^ Regulatory T cell (Treg) cell biology in juvenile idiopathic arthritis (JIA) and place this in the context of recent advances in understanding basic Treg biology. For review of JIA Treg biology, a defined Pubmed search was performed with the following terms: “Treg” OR “Foxp3” OR “Regulatory T cell” AND “Juvenile Idiopathic Arthritis”^1^. What is clear from these papers is that there has been great industry in elucidating Treg and effector T-cell biology and relating this to disease mechanisms from cellular viewpoints. This article aims to build on this body of knowledge by detailing how recent new approaches are giving fresh insight into the molecular control of *Foxp3* and the dynamics of T-cell regulation. It is hoped that this approach may help human immunologists to disambiguate markers used to identify Treg [e.g., CD25 and CD127 expression (1)] and stimulate fresh thinking about Foxp3-mediated regulatory mechanisms in JIA.

## Introduction

Foxp3 is essential for T-cell homeostasis and is considered one of the main drivers of Regulatory T-cell (Treg) differentiation (2). Mutations in the *FOXP3* gene in humans cause the complex multiorgan autoimmune disease, Immune Dysregulation Polyendocrinopathy Enteropathy X-linked syndrome (IPEX) (3). This condition parallels the Scurfy mouse (4), where a two base-pair insertion in the murine *Foxp3* gene results in a truncated form of Foxp3 protein (5) and loss of immune regulation. Scurfy mice typically die within 3 weeks, highlighting that loss of function of Foxp3 is not compatible with long-term survival. A Treg lineage (6) interpretation of these findings is that *Foxp3* mutations lead to loss of a dedicated line of suppressor T-cells, presumably because Foxp3 can no longer imprint the Treg suppressive phenotype (7). Treg are often divided into thymic and peripheral subsets, based on their sites of differentiation. Thymic Treg are self-reactive, and are important components of central tolerance. Peripheral Treg are thought to arise in response to innocuous antigens, and may be important for tolerance to dietary antigens or commensal bacteria. Together these subsets work to exert dominant tolerance to both self and foreign antigens (8).

Given the importance of Foxp3 to immune homeostasis and Treg biology, it has become a focal point of immunological research into diseases arising due to dysregulated T-cell responses, such as JIA. JIA is the most common form of autoimmune rheumatic disease with a prevalence in the region of one in a thousand in children under 16 years of age (9). JIA is a heterogeneous group of conditions, covering all forms of arthritis commencing in children under 16 years of age and lasting for at least 6 weeks duration (10). Although heterogeneity represents a challenge to translate basic immunological findings from animal models to JIA, access to the site of inflammation provides precious material for studying underlying immunological mechanisms.

## Genetics of JIA point to T-cell regulation

Genetic association studies of JIA (incorporating the most common subtypes of disease) clearly highlight a key role for genes involved in the immune system, particularly those involved in the regulation of T-cell biology (11). Unsurprisingly, the strongest association is with human leukocyte antigen (*HLA*) alleles. This is a common occurrence in autoimmune diseases (12), which may be due to the finding that self-antigen-specific Treg selection is HLA allele-dependent and modulates susceptibility to autoimmunity (13). There also exists a striking association with genes involved in the molecular control of Treg biology. Although nine single nucleotide polymorphisms (SNPs) within the human *FOXP3* gene show no significant associations to JIA (14) (suggesting these do not impact on *FOXP3* in JIA), a key *FOXP3*-binding partner, Runt-related transcription factor 1 (*RUNX1*), is, however, significantly associated. In non-Foxp3 expressing T-cells, RUNX1 enhances interleukin (*IL)2* expression through direct binding of the *IL2* promoter in activated CD4^+^ T-cells (7). However, in the context of Foxp3 expression, functional studies in mice have shown that Runx1 and Foxp3 form molecular complexes which lead to the repression of *Il2* and interferon gamma (*Ifng*), and upregulation of Treg effector molecules, such as CD25 and cytotoxic lymphocyte antigen 4 (CTLA-4) (7). Thus, Foxp3 can re-direct the molecular machinery involved in T-cell activation in order to drive a T-cell-intrinsic suppressive programme. Whilst Foxp3 expression has been mainly studied in the context of Treg generation (15), the T-cell-intrinsic functions of FOXP3 expression during either T-cell activation (which is considered transient, but can have functional consequences for effector T-cells 16) or peripheral (p)Treg generation in JIA remain unknown.

In addition to *RUNX1*, genes encoding proteins involved in cytokine signaling pathways that are critical to the development of FOXP3^+^ T-cells are also significantly associated. *IL2RA* [gene encoding CD25, the original Treg marker (17)] and the *IL2/IL21* cytokine locus display significant disease associations (11). Furthermore, deficiency in the IL-2 signaling molecule signal transducer and activator of transcription (STAT)5b has been reported in patients with JIA (18). IL-2 signaling is critical for the survival and fitness of Treg in the periphery (19) and is sensed by the conserved non-coding sequence 2 (CNS2) of the *Foxp3* gene to maintain Treg cell identity (20). These findings suggest that molecular tuning of *Foxp3* transcription and function not only have genetic associations but may represent a new avenue through which to further our understanding of the pathogenesis of JIA.

## Foxp3^+^ treg cell biology in JIA

Analysis of Treg cells identified by CD25 expression has shown that such cells are enriched at the site of inflammation in JIA (21). The authors further dissected whether CD25^+^ T-cells show any relationship to disease severity. Here analysis of the oligoarticular (O)-JIA subset of patients is very useful since the O-JIA patient group can have divergent clinical outcomes (22). The “persistent” O-JIA subtype presents with a mild, remitting form of disease, which can spontaneously resolve. However, there exists a subset of O-JIA patients in which the disease progresses and arthritis extends to include an increasing number of joints, which are referred to as extended O-JIA patients. These divergent forms of O-JIA have allowed translational immunologists to compare between different severities of disease, which has proven powerful for correlating the role of Treg and T-helper subsets in JIA with measurable disease outcomes. Interestingly, CD25^+^ Treg cells are present in increased numbers within the joints of JIA patients with the milder persistent form of O-JIA compared to those with the more severe form of extended O-JIA disease (21). These findings were further verified using the “gold standard” Treg marker, FOXP3, in a separate patient cohort (23). Interestingly, both of these studies highlighted that compared to Treg in blood, Treg at the inflamed site were not only increased in proportion but they also displayed increased FOXP3 protein expression at the single cell level (24). These seemingly paradoxical findings of a vastly increased Treg signature at inflamed sites suggest that Treg presence alone may not detail the whole picture. Indeed, many subsequent studies have highlighted that the balance of effector and regulatory mechanisms may be a major influencer of disease outcome. Evidence to support this in JIA comes from findings that Th17 cells, which drive chronic arthritis via a cellular cascade (25), are also increased in the synovial fluid (SF) of JIA patients and display a reciprocal relationship with Treg (23) or FOXP3 expression levels (24).

Alterations in Treg phenotypes are likely influenced by the joint environment (26, 27). Firstly, Treg from SF are susceptible to the downregulation of FOXP3 following removal from the inflamed environment (26), which can be prevented by addition of synovial fluid to cultures. Furthermore, analysis of thymic Treg output [which has been shown to be altered in adult rheumatic disease (28)] revealed that JIA patients are no different from controls (29), suggesting that the quantity of thymic Treg generation may be unaffected. Furthermore functional studies on Treg from JIA patients suggest no qualitative difference in their ability to suppress T-cell responses (30).

**Figure 1:**
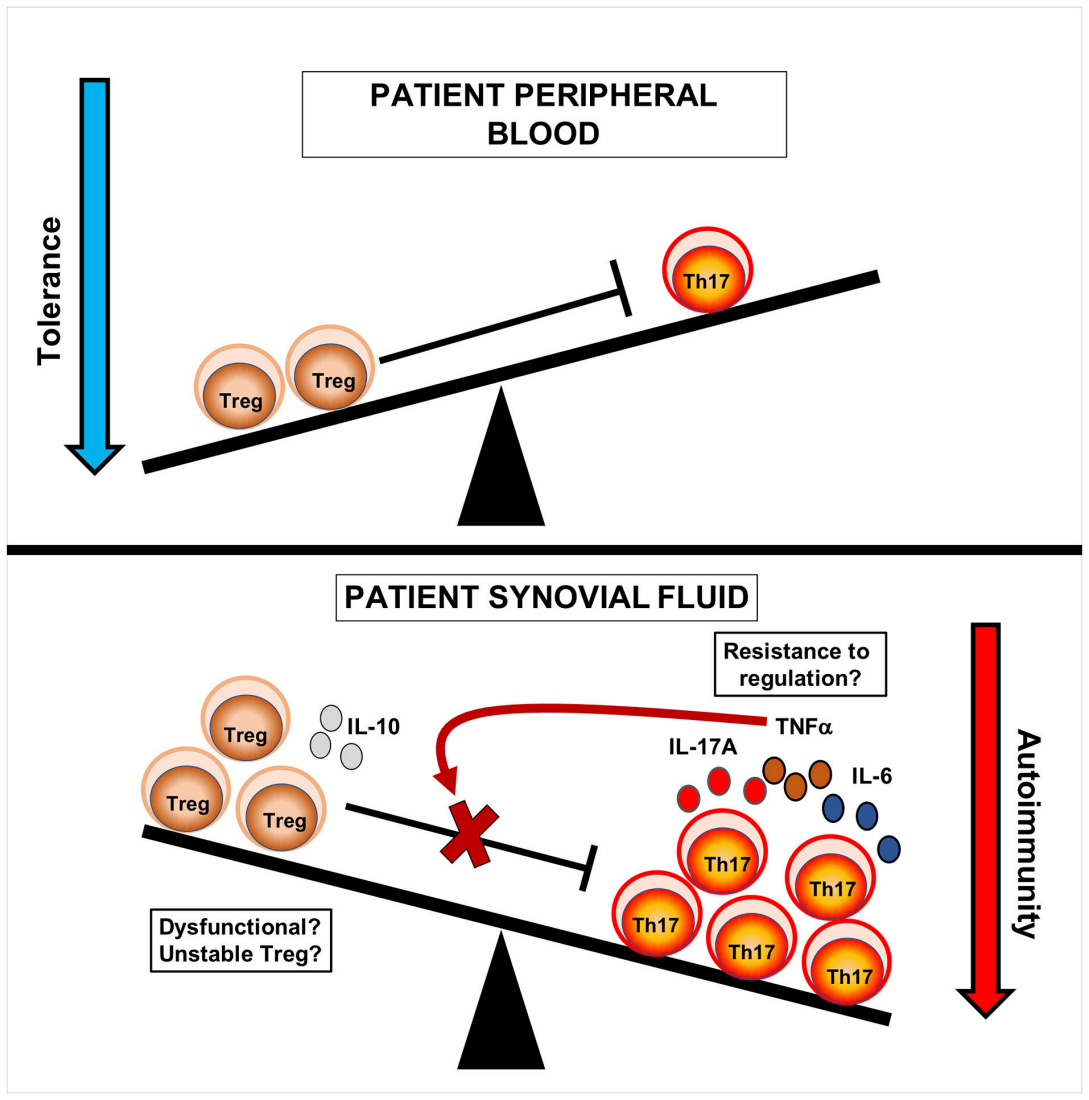
Cellular seesaw view of Treg and Th17 cells in JIA pathogenesis. In blood, immune relationships are balanced and Treg can regulate effector T-cells. In synovial fluid, Treg are increased at the inflamed site but show a reciprocal relationship to Th17 cells. This suggests that the balance of Th17 to Treg cells may influence disease course, likely through increased production of inflammatory mediators. Treg may be dysfunctional, either through loss of Foxp3 and lineage stability, or are unable to regulate Tconv at the site of inflammation due to the release of inflammatory cytokines such as TNFα. Current strategies have aimed to tip the balance of this system in order to promote tolerance over autoimmunity.

As to what may explain disease in the presence of increased Treg frequency, investigators have looked at the effector T-cells. *In vitro* suppression assays have shown that Treg from the blood and SF of JIA patients display similar abilities in regulating the proliferation of conventional T-cells (Tconv) from the peripheral blood environment (30, 31). However, Tconv from the inflamed site showed resistance to suppression by Treg. Neat follow up *in vitro* studies to this have gone on to show that this resistance to suppression could be overcome by the blocking of inflammatory cytokines such as tumor necrosis factor alpha (TNFα) (32, 33), which may provide a potential mechanism of action for biologics used to treat JIA. However, whilst these studies suggest that SF Tconv may be more resistant to regulation *in vitro*, it is unknown whether Tconv are resistant to regulation *in vivo*. In particular, analysis of Ki67^+^ (marker of cell proliferation) T-cells in the joints of JIA patients suggests a more complex picture, since the majority of dividing cells in the joint are Treg or Treg-like [as evidenced by hypomethylation of the Foxp3 gene (27)]. Nonetheless a consensus has built based on the aforementioned work that site-specific Treg and Tconv interactions play central roles in the pathogenesis of JIA (Figure 1).

## Dynamics and clonal relationships between Foxp3^+^ and Foxp3^−^ T-cells in JIA

Recent advances in sequencing technologies has allowed the profiling of the T-cell receptor (TCR) repertoires of Treg in JIA, which can be used to assess the relative clonal sharing between different CD4^+^ T-cell subsets and provide great insight into cellular dynamics. Deep sequencing of TCRβ chains has highlighted more restricted and oligoclonal repertoires in SF T-cells compared to blood (26, 34). Comparison between controls and JIA peripheral blood Treg suggests that JIA patients have skewing of both blood and SF TCR repertoires (34). This skewing of the repertoire could arise from recirculation of T-cells between blood and SF (35), but potentially could be an important biomarker for treatment. Indeed, animal studies have shown that bone marrow transplantation following peptidoglycan-induced arthritis revealed that Treg from the graft repopulate the immune system and show improved repertoire diversity (36). In fact, transfer of additional Foxp3-GFP^+^ T-cells together with the bone marrow transfer graft did not induce additional clinical improvement but moreover delayed TCR repertoire diversification, cautioning the use of Treg transfers in such settings. This is likely due to the fact that these additional Treg suppress T-cell proliferation, thus delaying expansion of donor-derived T-cells. In addition, the authors were able to show that JIA patients undergoing haematopoietic stem cell transfers also showed improved Treg diversity. These findings suggest that an immune reset provides an opportunity for graft-derived cells to regulate the autoimmune response, likely due to alterations in T-cell dynamics. Indeed, the SF environment appears to drive dynamic changes in Treg expression of key molecules. TCR repertoire analysis of SF T-cells expressing different combinations of CD25 and FOXP3 have shown that there is remarkable clonal sharing between CD25^+^FOXP3^−^, CD25^+^FOXP3^+^, and CD25^−^FOXP3^+^ T-cells within the joint (26). These findings suggest that CD25 and FOXP3 expression within Treg-like populations may be dynamic within the joint; however, this does not preclude the possibility that CD25+FOXP3- T-cells also contain recently activated effector T-cells. Indeed, a thorough dissection of these populations would give useful insight to the dynamics of T-cell activation and FOXP3 expression in JIA. Given this observation, it will be intriguing for future studies to interrogate relationships between the TCR repertoires of Th17 and Treg cells within the joints of O-JIA patients, in order to establish whether the reciprocal relationship arises from discrete clones, or whether Th17/Treg plasticity is an important mechanism. For instance hybrid IL-17A^+^ FOXP3^+^ Treg, identified by expression of CD161, are significantly enriched at the site of inflammation (37, 38), and repertoire overlap between CD161^+^ Treg and Tconv as a proportion of CD161^+^ SF Treg was in the region of 20–30% (38), suggesting plasticity of T-cell responses within the joint. In summary, what these papers clearly show is that the joint environment is highly dynamic, and that snapshot in time analyses may obscure observations that may be accounted for by cellular and molecular dynamics.

## Molecular dynamics regulate Foxp3+ T-cell biology

**Figure 2:**
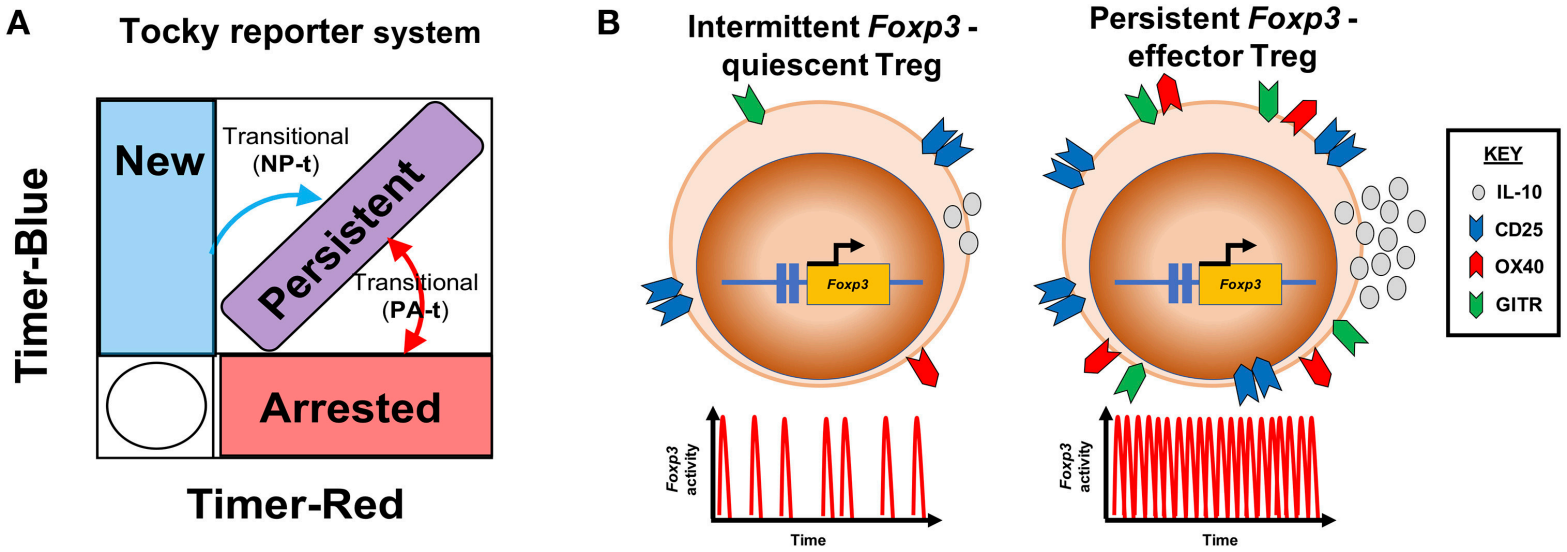
Transcriptional dynamics of *Foxp3* define functional Treg profiles. **(A)** The Tocky system [adapted from (39)] identifies different temporal expression patterns of the *Foxp3* gene. Here a schematic is shown, illustrating how the Tocky system's timer locus approach can be used to identify different dynamics of *Foxp3* expression based on the position of cells within a theoretical flow cytometric Timer-blue and Timer-red two-dimensional space: New expressers are pure blue, gradually acquiring red fluorescence after 4–8 h, and moving to the New-Persistent transitional (NP-t) locus. Cells which continually transcribe *Foxp3*, accumulate in the blue^+^ red^+^ diagonal, called Persistent. And cells that have recently ceased *Foxp3* transcription are located in the pure red Arrested locus. As *Foxp3* gene activity changes, cells can move between Arrested and Persistent zones within the Persistent-Arrested transitional (PA-t) locus. **(B)** Summary of Treg *Foxp3* gene dynamics under different immunological contexts. In steady state, Treg exhibit intermittent *Foxp3* gene activity, and express lower levels of CD25, GITR, OX40, and IL-10. Upon immune challenge, responsive Treg increase their *Foxp3* transcriptional activity to a temporally persistent dynamic, which drives the Treg effector functions.

Whilst Foxp3 expression is used to define Treg subsets and is considered a stable marker of Treg cells, the clonal relationships between Treg and Tconv by TCR sequencing can also be explained by taking a molecular perspective (6). For instance, we have recently generated a new reporter system called Timer of cell kinetics and activity (Tocky) [(39); Figure 2A], which highlights a feedback control role for Foxp3 in regulating T-cell responses. This approach places the emphasis on the molecular dynamics of the system, with a reduced focus on cellular categorization, which can unnecessarily constrain analysis, particularly for high-dimensional data sets.

## Timer of cell kinetics and activity (tocky) system

Tocky reporter system uses a short-lived fluorescent Timer protein (40) to capture the activity of the *Foxp3* gene. Timer protein exhibits an initial blue fluorescent form, with an approximate half-life of 4 h in *Foxp3*-Tocky mice. Timer protein undergoes spontaneous and irreversible maturation into a red fluorescent form which has a half-life in the region of 5 days (41). *Foxp3*-Tocky therefore allows the detection of biologically important *Foxp3* gene settings. New *Foxp3* expressers are identified in *Foxp3*-Tocky mice by virtue of their pure blue fluorescence upon expression of the Timer gene, which is under the control of the *Foxp3* gene regulatory elements. This system captures rapid changes in *Foxp3* gene settings in response to immunological cues, giving insight to *Foxp3* gene regulation at the level of hours vs. days. In contrast, fate-mapping approaches, which have largely suggested at a cellular level Treg cells are stable (42, 43), do not capture the “real-time” changes in *Foxp3* gene activity which may greatly influence Treg function (41).

## Foxp3-tocky reveals effector treg differentiation and identifies pTreg

We have revealed that mature Foxp3 expressers tune their *Foxp3* gene setting to a temporally persistent state to control the resolution of skin inflammation. Thus, our recent data suggest that studying Foxp3 as a binary marker (i.e., to identify Treg and non-Treg) can lose biological information. Upon immunization we observed dramatic changes in the activity of *Foxp3* transcription in various T-cell populations. At sites of inflammation (which are the relevant comparison for JIA) Treg increase transcription of *Foxp3* in a Foxp3-protein dependent fashion (41). This form of *Foxp3* autoregulation is key to driving what has been previously called the effector (e)Treg response, where cells display enhanced expression of immunoregulatory molecules, such as IL-10 and CTLA4. Thus, purely based on a molecular readout—the activity of the *Foxp3* gene—we could identify the major features of previously coined “quiescent” Treg and effector (e)Treg subsets (Figure 2).

Foxp3-Tocky mice allow visualization of the earliest stages of p Treg development during physiological T-cell responses. We reported an increased proportion of T-cells acquire *de novo* Foxp3 expression within inflamed skin compared to non-inflamed sites, during contact hypersensitivity. The biological significance of this is still to be fully determined, but we propose that induction of Foxp3 may be an important part of the resolution of T-cell responses through the intrinsic regulation of Tconv (16, 41).

## Post-translational control of foxp3

Aside from the transcriptional control of *Foxp3*, insight into the post-translational control of Foxp3 protein has been revealed over the past few years (44). Foxp3 protein has been shown to be polyubiquitinated at multiple lysine residues, which can lead to its proteasome-mediated degradation (44, 45). This process appears to be regulated by the activity of two enzymes, the deubiquitinase USP7 (44) and E3 ubiquitin ligase STUB1 (45). Here the authors of the studies were able to show that inflammatory cytokines, such as IL-6 [which is elevated in JIA synovial fluid (46)] could repress USP7 expression, resulting in increased turnover of Foxp3 and loss of Treg control of inflammation. These findings have led to the suggestion that small molecular inhibitors of this process could improve the stabilization of Foxp3 protein in cells, and therefore their functions (47).

## Foxp3 molecular “tuning” for therapy

Foxp3^+^ T-cells have a large number of molecules that have been proposed as surrogate markers, such as high CD25 expression and low CD127 (1) (the IL-7 receptor alpha chain). In addition, they have also been shown to express high levels of the Tumor necrosis factor receptor superfamily members, which are important for their thymic development (48). Indeed, it has been known for over 15 years that glucocorticoid-induced TNFR-related protein (GITR) is a marker of thymic CD4^+^ CD25^+^ subsets and targeting this membrane receptor with a monoclonal antibody could alter the course of autoimmunity (49). Using the *Foxp3*-Tocky tool, we have been able to give a dynamic perspective to common Treg cell surface markers (41). We were able to classify membrane receptors according to their relationship to *Foxp3* transcriptional activity, revealing that they can be classified into two main groups. Group I [containing amongst others TNFRII, C-C chemokine receptor type four (CCR4), CCR5] are high on activated T-cells and new *Foxp3* expressers and remain high whilst active *Foxp3* transcription occurs. We could show that the targeting of a marker within this group (TNFRII) was able to increase the proportion of T-cells acquiring new *Foxp3* expression. Group II (containing amongst others CD25, OX40, and GITR) membrane receptors appeared to parallel the activity of *Foxp3* transcription and increased as T-cells moved into persistent dynamics of *Foxp3* Transcription (i.e., eTreg phenotype). Very interestingly, expression levels of these molecules fell considerably in T-cells with low or arrested *Foxp3* transcriptional activity, implying they are selective to the eTreg-type programme. OX40 was one member of this group, and we showed that upon anti-OX40 treatment Foxp3^+^ T-cells were shorter lived and “persistent” Foxp3 transcribers were reduced. This correlated with a delay in the resolution of allergic T-cell driven skin inflammation (41). Although these effects were modest, and the precise mechanisms of action remain to be fully elucidated, they show proof of concept that *Foxp3* transcriptional dynamics within T-cell populations can be modulated with measurable changes in disease outcomes.

## Conclusions and future directions

This review has highlighted the increases in our understanding of Foxp3^+^ T-cell biology in JIA. Recent work revealing how molecular pathways regulate Foxp3 protein and *Foxp3* transcription should spur researchers to consider how these findings may be translated to human disease settings. For instance, it will be useful to determine whether the markers identified in basic animal models also hold equally true for humans. This could provide biomarkers to better understand the immunological effects of biologics for the treatment of autoimmune disorders such as JIA. In addition, given that Foxp3 protein can autoregulate its transcription, it will be very interesting to see whether post-translational modifications of Foxp3 can alter the Foxp3-driven autoregulatory loop (41, 50), and therefore enhance the Foxp3-driven T-cell programme. To take these ideas forward, however, the field may need to take a few steps back from the cell lineage paradigm and consider how the molecular dynamics of Foxp3-driven biology may provide new avenues for translational research in JIA.

## Author contributions

DB conceived the review. AC and DB reviewed the literature and co-wrote the manuscript.

### Conflict of interest statement

The authors declare that the research was conducted in the absence of any commercial or financial relationships that could be construed as a potential conflict of interest.

## Footnotes

^1^Search date July 10^th^ 2018.
